# Tight-binding model for opto-electronic properties of penta-graphene nanostructures

**DOI:** 10.1038/s41598-018-29288-8

**Published:** 2018-07-23

**Authors:** Sergio Bravo, Julián Correa, Leonor Chico, Mónica Pacheco

**Affiliations:** 1 Universidad Técnica Federico Santa María, Departamento de Física, Valparaíso, Casilla 110-V Chile; 2 Universidad de Medellín, Facultad de Ciencias Básicas, Medellín, Colombia; 3 Materials Science Factory, Instituto de Ciencia de Materiales de Madrid (ICMM), Consejo Superior de Investigaciones Científicas (CSIC), C/ Sor Juana Inés de la Cruz 3, 28049 Madrid, Spain

## Abstract

We present a tight-binding parametrization for penta-graphene that correctly describes its electronic band structure and linear optical response. The set of parameters is validated by comparing to ab-initio density functional theory calculations for single-layer penta-graphene, showing a very good global agreement. We apply this parameterization to penta-graphene nanoribbons, achieving an adequate description of quantum-size effects. Additionally, a symmetry-based analysis of the energy band structure and the optical transitions involved in the absorption spectra is introduced, allowing for the interpretation of the optoelectronic features of these systems.

## Introduction

The discovery of graphene has stimulated the quest for novel two-dimensional (2D) materials, resulting in the experimental synthesis and the theoretical prediction of various layered systems with diverse properties^1–3^. Besides elemental analogs of graphene, such as silicene, germanene or stanene, hexagonal 2D crystals such as boron nitride or transition-metal dichalcogenides are the focus of intense research, both applied and fundamental^4^. Many of these materials can be obtained by mechanical exfoliation of a three-dimensional crystal composed of weakly interacting layers coupled by van der Waals forces, like graphene itself. In fact, crystals with weakly bonded layers are being examined as a source of new bidimensional materials. Beyond mechanical methods, chemical exfoliation techniques have also been applied to covalently bonded layers to produce such 2D materials^5,6^.

Inspired by such techniques, it has been theoretically proposed that penta-graphene (PG), a new 2D carbon allotrope, can be obtained from T12-carbon by breaking the covalent bonds between layers^7^. Although PG is a metastable carbon allotrope compared to graphene, it is energetically more favorable than the icosahedral fullerene C_20_ or the smallest nanotube, which have been synthesized. So despite some claims regarding its instability^8,9^, it is reasonable to expect that PG might be experimentally viable. Analogously to graphene, PG could be encapsulated by hexagonal boron nitride (hBN); this could help to achieve stability, besides providing insulation from chemical agents^10,11^.

Penta-graphene has been predicted to possess several unique characteristics. It is not completely planar, and it does not have a hexagonal lattice; its 2D projection is the Cairo tiling, being composed of fused pentagons. From the electronic viewpoint, it is a semiconductor with a quasi-direct bandgap^7^, being attractive for optoelectronic applications. PG has a reduced thermal conductivity compared to graphene^12–14^ and it is an auxetic material, i.e., it has a negative Poisson’s ratio^7,15^. It has been proposed as an anode material in alkaline batteries^16^, as a metal-free catalyst for CO oxidation^17^ and for use in hydrogen storage systems^18^. Furthermore, by doping and functionalization, the mechanical, optical, and electronic properties of PG can be tuned. Hydrogenation and fluorination of PG have also been analyzed, with focus on the consequences in the band gap variation^19–21^.

As in other 2D systems, the properties of nanostructures with lower dimensions based in PG have been also explored. For example, PG nanoribbons^7,22–24^ multilayer PG^9,25^ and PG nanotubes^7,9,26^, which might be even more stable than monolayer PG. Most of these works employ a first-principles approach; recently, a tight-binding (TB) model has been put forward, allowing for the obtention of the electronic bands and an analytical expression for the optical absorption^27^. In fact, Zhang *et al*. also provided a minimal tight-binding parameterization in their pioneering work, but with a limited agreement to the ab-initio bands^7^.

The aim of this work is to present a tight-binding parameterization of penta-graphene with an emphasis in the quantitative description of its optical spectrum, valid also for wide PG nanoribbons for which ab-initio calculations are costly. We choose an edge termination that does not make the ribbons magnetic, in order to focus on size effects in these systems. While ARPES measurements provide a direct comparison to the electronic bands, optical spectra are a standard characterization tool that is crucial for the identification of semiconductor materials, hence our motivation for this approach. We obtain our parameters by a fit to the ab-initio bands, checking that the resulting parameters give a good description of the optical absorption in PG. Additionally, we perform a symmetry analysis of the band structure and the optical spectra of these systems.

The paper is organized as follows. In section 2 we describe the lattice structure and symmetry of the system and the computational methods. In section 3 we explain the tight-binding parameterization and present our results for the electronic structure of a monolayer PG computed with this model, and compare it with the ab-initio bands. In the same section we also show the optical absorption response of PG and penta-graphene nanoribbons calculated with the tight-binding model, along with the corresponding ab-initio result. Finally in section 4, we finish with some conclusions.

## Geometry and Computational Methods

### Penta-graphene lattice geometry

**Figure 1:**
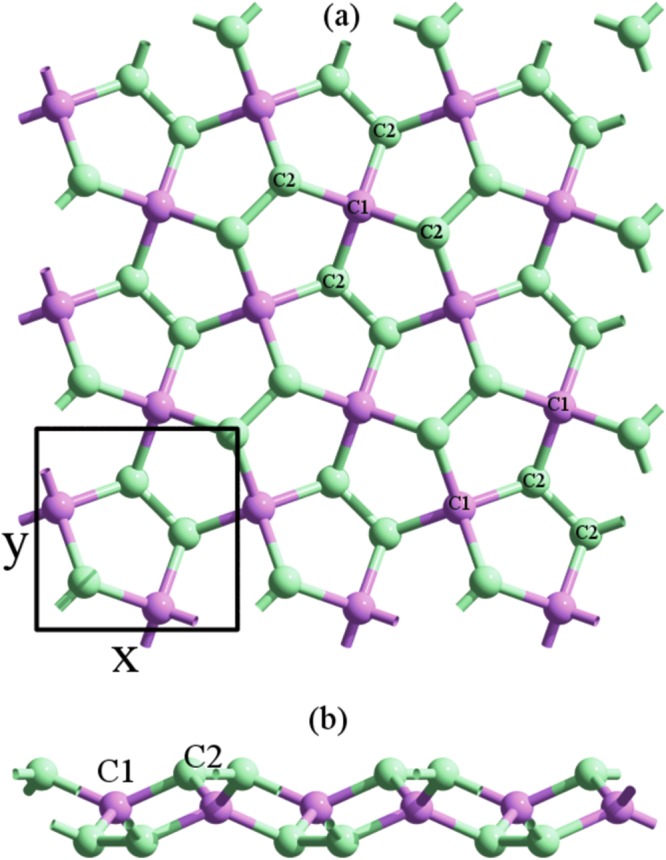
(**a**) Top and (**b**) side views of the PG lattice. The unit cell comprising 6 carbon atoms is enclosed in a black square. Atoms with coordination number 4 are labeled as C1, those with coordination number 3 are labeled C2.

As described by Zhang. *et al*.^7^, penta-graphene has a buckled lattice structure composed by non-planar carbon pentagons, shown in Fig. 1. The space group of this crystal lattice is P$\overline{4}$2_1_m (#113)^28,29^, which is nonsymmorphic. The unit cell has six carbon atoms, highlighted with a black box in Fig. 1(a). The buckled lattice structure of PG can also be described as composed of three layers, see Fig. 1(b). Notice that two of the atoms in the unit cell, labeled C1, have coordination 4. They belong to the central layer, whereas the other four C2 atoms have coordination 3 and form the outer layers of PG. The difference in coordination number is obviously related to a different hybridization: C1 atoms have a *sp*^3^ character, whereas C2 atoms are more *sp*^2^-like.

### Computational methods

Since the tight-binding model requires the adjustment of empirical parameters, it is necessary to resort to *ab*-*initio* results in order to fit their values, given that for the time being, no experimental data are available. The optical absorption is calculated within the electric dipole approximation in both approaches.

#### Ab-initio approach

We employ the Density Functional Theory (DFT) approach, using the SIESTA ab-initio code^30^, to calculate the opto-electronic properties of monolayer PG and penta-graphene nanoribbons (PGNR). In particular, we use for the exchange-correlation functional the generalized gradient approximation (GGA) of Perdew-Burke-Ernzerhof^31^ instead of more expensive hybrid functionals^7^, because the observed general trends in the electronic structure remain almost unaltered in both schemes, and only the magnitude of the band gap is changed^7,27^. We use a double-*ζ* plus polarization basis set and norm-conserving pseudopotentials. The mesh cutoff was set to 150 Ry and the energy shift to 0.07 eV. The Brillouin zone was sampled with a 15 × 15 × 1 Monkhorst-Pack grid for PG and a 10 × 1 × 1 Monkhorst-Pack grid for PGNRs. A conjugate gradient self-consistent procedure was used to relax all structures with a maximal force tolerance per atom of 0.04 eV/Å. These sets of parameters assured a good energy convergence. In the case of PGNR, edges were passivated with hydrogen in order to saturate the dangling bonds.

For the optical absorption, we use a 201 × 201 × 1 k-point grid for PG and a 201 × 1 × 1 k-point grid for the PGNRs, with a 0.06 eV broadening for both structures. We also assume that the electromagnetic (EM) radiation is incident perpendicularly to the PG sheet, i.e., with the electric field **E** polarization fixed in the *xy* plane.

#### Tight-binding model

We follow the Slater-Koster (SK) approach^32^ for orthogonal tight-binding calculations with the aim of providing the simplest model with a good description of the electronic and optical properties. Our first concern is the orbital basis choice. Penta-graphene only has carbon atoms, so we take the usual basis selection of one *s* orbital and three *p* orbitals per atom. There are 6 atoms in the unit cell of PG; thus our basis for the SK Hamiltonian has 24 orbitals. Note that previous parameterizations with fewer orbitals present a poor agreement with DFT bands; only consideration of the full *sp*^3^ basis provides a reasonable accord^27^. Our goal is to find a set of parameters which gives not only a good depiction of the band structure, but also of the optical properties, so nanostructures based in PG, such as nanoribbons and nanotubes could be described within this approach in a computationally affordable manner.

Within the electric dipole approximation, the optical absorption coefficient is given by

1 $$\alpha (\omega )=\frac{4{\pi }^{2}{e}^{2}}{{n}_{0}c\,{m}^{2}\omega }\,\sum _{c,v,{\bf{k}}}\,|{P}_{cv}({\bf{k}}){|}^{2}\delta ({E}_{c}({\bf{k}})-{E}_{v}({\bf{k}})-\hslash \omega ),$$

where *e* and *m* are the charge and mass of the electron respectively, *ω* is the frequency of the EM radiation, *n*_0_ is the refraction index, *c* is the velocity of light (here set to the vacuum values) and *P*_*cv*_(**k**) are the electric dipole matrix elements ${P}_{cv}=\langle c,{\bf{k}}|{\bf{u}}\cdot {\bf{r}}|v,{\bf{k}}\rangle ={\bf{u}}\cdot \langle c,{\bf{k}}|{\bf{r}}|v,{\bf{k}}\rangle$, where **u** is the polarization vector of the external electric field and **r** is the vector position operator, evaluated between eigenstates |*v*, **k**〉 and |*c*, **k**〉 of the valence (*v*) and conduction (*c*) bands with eigenenergies *E*_*v*_(**k**) and *E*_*c*_(**k**), respectively.

In order to calculate the absorption coefficient within the TB approximation, we express the matrix elements of the position operator in terms of the tight-binding parameters. In line with the procedure given in refs^33–35^, we use the following identity for the position operator matrix,

2 $$\langle c,{\bf{k}}|{\bf{r}}|v,{\bf{k}}\rangle =\frac{1}{{E}_{c}({\bf{k}})-{E}_{v}({\bf{k}})}\langle c,{\bf{k}}|[{\bf{H}},{\bf{r}}]|v,{\bf{k}}\rangle ,$$

where *H* is the unperturbed Hamiltonian of the system. Expanding the eigenstates of *H* into a linear combination of atomic orbitals $|n,{\bf{k}}\rangle ={\sum }_{i}\,{C}_{n}(i)|i,{\bf{k}}\rangle$, where *n* is the band index, *i* is the atomic orbital index and *C*_*n*_ (*i*) are the expansion coefficients, we obtain the following expression for the dipole matrix elements *P*_*cv*_,

3 $${P}_{cv}={\bf{u}}\cdot \sum _{i,j}\,\frac{{C}_{c}^{\ast }(i){C}_{v}(j)\,{\sum }_{{{\bf{R}}}_{ij}}\,{{\bf{R}}}_{ij}{e}^{{\bf{ik}}\cdot {{\bf{R}}}_{ij}}{t}_{ij}({{\bf{R}}}_{ij})}{{E}_{ck}-{E}_{vk}},$$

where **R**_*ij*_ are the lattice vectors and *t*_*ij*_ (**R**_*ij*_) represent the Slater-Koster TB parameters. With this expression, we can compute the optical absorption coefficient shown in Eq. (1).

## Results

### Tight-binding parameterization

Since we follow the Slater-Koster scheme we have to assign to the orbital integrals the corresponding parameters. For PG it is already known that a simple scaling of a graphene-based parameterization yields a qualitative agreement, and a fit to DFT bands is needed to improve this description^27^. With this purpose we analyze the bonding structure and geometry of PG. As discussed in the previous Section, in the 6-atom unit cell of PG there are two carbon atoms with coordination 4 and *sp*^3^ hybridization, i.e., with four bonds each, labeled C1, and four carbon atoms with three bonds each, labeled C2, with *sp*^2^ character. This partition leads us to treat each group of atoms separately with respect to the SK parameterization. The basic idea is that these two groups of atoms not only have different nearest-neighbor (NN) distances, but also different hybridizations. From Fig. 1 we see that the first NNs for C1 atoms are four C2 atoms. In turn, the C2 atoms only have one NN, a C2 atom which is in the same layer. Therefore, we can assign a group of first NN parameters for each group. We parameterize the C1-C2 interaction with the SK integrals ${V}_{ss\sigma }^{C1C2}$, ${V}_{sp\sigma }^{C1C2}$, ${V}_{pp\sigma }^{C1C2}$, ${V}_{pp\pi }^{C1C2}$, and the C2-C2 interaction with integrals ${V}_{ss\sigma }^{C2C2}$, ${V}_{sp\sigma }^{C2C2}$, ${V}_{pp\sigma }^{C2C2}$, ${V}_{pp\pi }^{C2C2}$. On the other hand, for the C2 atoms we already have included up to second NNs. We consider also the hoppings between a C2 carbon atom in one of the external planes and another C2 atom from the opposite one, which we have labeled as C2′ to distinguish it from the first NN C2-C2 pair. This interaction is indeed a third NN interaction, and we can assign the corresponding SK integrals ${V}_{ss\sigma }^{C2C2^{\prime} }$, ${V}_{sp\sigma }^{C2C2^{\prime} }$, ${V}_{pp\sigma }^{C2C2^{\prime} }$, ${V}_{pp\pi }^{C2C2^{\prime} }$.

This exhausts the basic interactions for our model. We have checked that considering the next NN for the C1 atoms, i.e., another C1-C2 coupling, does not improve appreciably our results, so in fact we have a geometrical cutoff that includes interactions up to distances equal or smaller that the 3rd NN interactions between C2 atoms.

In summary, we have twelve SK hopping parameters with contributions up to first NN for the C1 atoms and up to third NN for the C2. Finally, we consider the onsite energies associated with each atom and orbital. Specifically, we assign four onsite energies, ${E}_{s}^{C1},\,{E}_{p}^{C1},\,{E}_{s}^{C2},\,{E}_{p}^{C2}$ corresponding to the *s* orbital and the three *p* orbitals for each group of atoms respectively. This amounts to a total of sixteen SK parameters in our model.

The bands are usually fitted at the high symmetry points and special lines, which are in principle the main contributions to the optical absorption. Such fitting is done with respect to the bands obtained within the DFT approach. However, we have verified that the four bands closer to the gap may have a very good agreement with the DFT bands, but without achieving a similarly acceptable description of the optical absorption. We have found that there are local maxima and minima in the dispersion relations, especially in the valence band, which cannot be fitted with this reduced set of parameters. We have explored several parameterizations along these special lines, and despite obtaining very good fits to the conduction bands, all fail to give the shape of the valence bands, which are relevant to the fit of the optical spectrum. Since the optical properties depend on all the states over the Brillouin zone (BZ), not only over the special lines, we decided to perform a fit to the energy dispersion relation over the entire 2D BZ, with 8000 *k* points. However, this did not improve the optical absorption results obtained by our initial method (See Supplementary Information). Therefore, it is necessary to further correct the parameter set considering the optical absorption in order to describe optimally both, the electronic structure and the optical response. Details of our computational procedure are described in the Supplementary Information. The final values for the TB parameters are presented in Table 1.

**Table 1:** Slater-Koster tight-binding parameters (in eV) for PG.

				${{\boldsymbol{E}}}_{{\boldsymbol{s}}}^{{\boldsymbol{C}}{\bf{1}}}$	${{\boldsymbol{E}}}_{{\boldsymbol{p}}}^{{\boldsymbol{C}}{\bf{1}}}$	${{\boldsymbol{E}}}_{{\boldsymbol{s}}}^{{\boldsymbol{C}}{\bf{2}}}$	${{\boldsymbol{E}}}_{{\boldsymbol{P}}}^{{\boldsymbol{C}}{\bf{2}}}$				
				−6.433	−4.311	−2.081	6.506				
${{\boldsymbol{V}}}_{{\text{ss}}{\boldsymbol{\sigma }}}^{{\boldsymbol{C}}{\bf{1}}{\boldsymbol{C}}{\bf{2}}}$	${{\boldsymbol{V}}}_{{\text{sp}}{\boldsymbol{\sigma }}}^{{\boldsymbol{C}}{\bf{1}}{\boldsymbol{C}}{\bf{2}}}$	${{\boldsymbol{V}}}_{{pp}{\boldsymbol{\sigma }}}^{{\boldsymbol{C}}{\bf{1}}{\boldsymbol{C}}{\bf{2}}}$	${{\boldsymbol{V}}}_{{pp}{\boldsymbol{\pi }}}^{{\boldsymbol{C}}{\bf{1}}{\boldsymbol{C}}{\bf{2}}}$	${{\boldsymbol{V}}}_{{\text{ss}}{\boldsymbol{\sigma }}}^{{\boldsymbol{C}}{\bf{1}}{\boldsymbol{C}}{\bf{2}}}$	${{\boldsymbol{V}}}_{{\text{sp}}{\boldsymbol{\sigma }}}^{{\boldsymbol{C}}{\bf{1}}{\boldsymbol{C}}{\bf{2}}}$	${{\boldsymbol{V}}}_{{pp}{\boldsymbol{\sigma }}}^{{\boldsymbol{C}}{\bf{2}}{\boldsymbol{C}}{\bf{2}}}$	${{\boldsymbol{V}}}_{{pp}{\boldsymbol{\pi }}}^{{\boldsymbol{C}}{\bf{2}}{\boldsymbol{C}}{\bf{2}}}$	${{\boldsymbol{V}}}_{{ss}{\boldsymbol{\sigma }}}^{{\boldsymbol{C}}{\boldsymbol{^{\prime} }}2{\boldsymbol{C}}{\bf{2}}}$	${{\boldsymbol{V}}}_{{sp}{\boldsymbol{\sigma }}}^{{\boldsymbol{C}}{\boldsymbol{^{\prime} }}2{\boldsymbol{C}}{\bf{2}}}$	${{\boldsymbol{V}}}_{{pp}{\boldsymbol{\sigma }}}^{{\boldsymbol{C}}{\boldsymbol{^{\prime} }}2{\boldsymbol{C}}{\bf{2}}}$	${{\boldsymbol{V}}}_{{pp}{\boldsymbol{\pi }}}^{{\boldsymbol{C}}{\boldsymbol{^{\prime} }}2{\boldsymbol{C}}{\bf{2}}}$
−3.555	2.246	3.903	−0.262	−11.731	−10.017	15.490	−1.762	−2.504	1.080	−3.247	−0.921

### Electronic properties of PG

**Figure 2:**
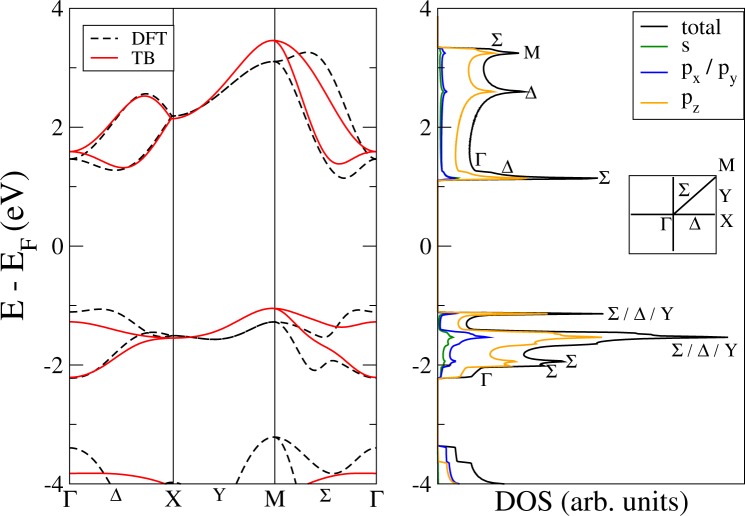
Left panel: PG energy band structures near the Fermi level calculated with DFT (black dotted lines) and tight-binding (red solid lines). Right panel: DFT-calculated penta-graphene total DOS (black solid line), and its decomposition in *s*-orbital (green), *p*_*x*_/*p*_*y*_-orbitals (blue) and *p*_*z*_ orbital (orange) projected DOS.

In the left panel of Fig. 2 we present the TB band structure obtained with the parameters given in Table 1 along with the bands calculated using the SIESTA code for PG. It can be seen that there is a very good overall agreement between them. We have been able to reproduce the conduction band minimum along the Σ line, among other features.

As mentioned before, it is feasible to obtain an excellent fit to the four bands of interest, especially the conduction bands, with the same number of parameters, but failing to reproduce other valence bands at lower energy. In particular, the maxima and minima of the valence bands in the Σ direction cannot be reproduced with this model. This *wiggling* of the valence bands also appears in other low-symmetry points of the BZ inside the 2D region enclosed by the special symmetry lines. We have found that the appearance of local maxima and minima in the DFT calculation, specially in the valence bands, is the reason why the optical spectrum is not even qualitatively correct (See Supplementary Information). We opted for a compromise solution, maintaining the overall agreement of the bands but without losing the description of the optical properties while keeping the same number of parameters.

Our election of tight-binding parameters can be supported with the orbital decomposition of the density of states (DOS) for monolayer PG, shown in the right panel of Fig. 2. The DOS calculation was performed with SIESTA with a dedicated 251 × 251 × 1 k-point grid for this particular calculation along with a energy broadening of 0.010 eV. The figure also shows the orbital decomposition of the DOS in *s* and *p* orbitals. As expected^27^, these four bands mainly have a *p* character, more specifically *p*_*z*_, albeit with a non-negligible contribution of the *p*_*x*_ and *p*_*y*_ orbitals, which are equivalent. In addition, we have identified the DOS peaks corresponding to high symmetry points and lines within the BZ, shown in the inset of the Figure, with the aim to elucidate the symmetry of the BZ points with a high density of states. This can serve as a guide to understand the optical features of the material.

### Optical properties of monolayer PG

**Figure 3:**
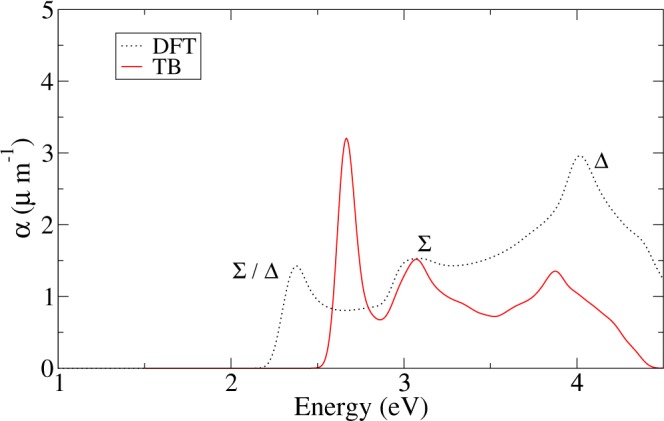
Optical absorption for PG calculated with tight-binding (red solid lines) and ab-initio SIESTA code (dashed black lines).

As in the case of the electronic band structure, we compare the optical absorption coefficient computed within the DFT approach with that calculated with the tight-binding approximation. In this case we use the same *k*-space grid and broadening energy in both calculations, TB and DFT. The results are shown in Fig. 3. There are three marked peaks in the low energy region of the DFT optical spectrum. We have labeled them according to the symmetry of the relevant states, as in the DOS plot. The lowest peak (~2.45 eV), is due to transitions near the band gap; it is dominated by contributions from a region around the Δ line, where the conduction and valence band states have an energy difference ~2.5 eV. The next peak (~3 eV) has contributions from the Σ line across the BZ, where many transitions are allowed due to the low symmetry. The last and higher peak in this energy window (~3.8 eV), can be related to the Δ line, where we have several allowed transitions due to the low symmetry present in this line, similar to those from the Σ direction and the peak near 3 eV. Additionally, we have checked that the Γ point does not have a major weight in this peak; its height is due to other low-symmetry points involving the local maxima and minima of the valence bands. Additional symmetry analysis gives us another interesting property related to the optical response of PG. Due to the group of the wave vector at the high symmetry points X and M of the BZ, we obtain a selection rule that forbids transitions from the valence to the conduction band at these two points. This is because of the different parities of the irreducible representations that are coupled by the momentum operator, giving a direct product that does not contain the invariant irreducible representation of the group^36^. Moving away from these points, the restriction is relaxed; the shoulder at ~4 eV has contributions of transitions from the Σ and Y lines around M and neighboring low-symmetry points.

The TB parameterization of Table 1 reproduces the main features in the optical absorption spectrum, as shown in Fig. 3. The two higher energy peaks, at 3 and 3.8 eV, appear at the same energy; the lower peak is blue-shifted around 0.3 eV. However, the intensities are not well described by the TB model. The lower and higher energy peaks show an appreciable difference in intensity compared to the DFT result. The central peak does match the DFT intensity. This is due to the fact that these two peaks stem from transitions involving the local maxima and minima of the valence bands, difficult to fit with this TB basis set. We would like to emphasize that our parameterization of the energy band structure, which uses the optical absorption as a criterion for its validity, manages to provide a remarkably good description of both features (See Supplementary Information).

### Penta-graphene nanoribbons

**Figure 4:**
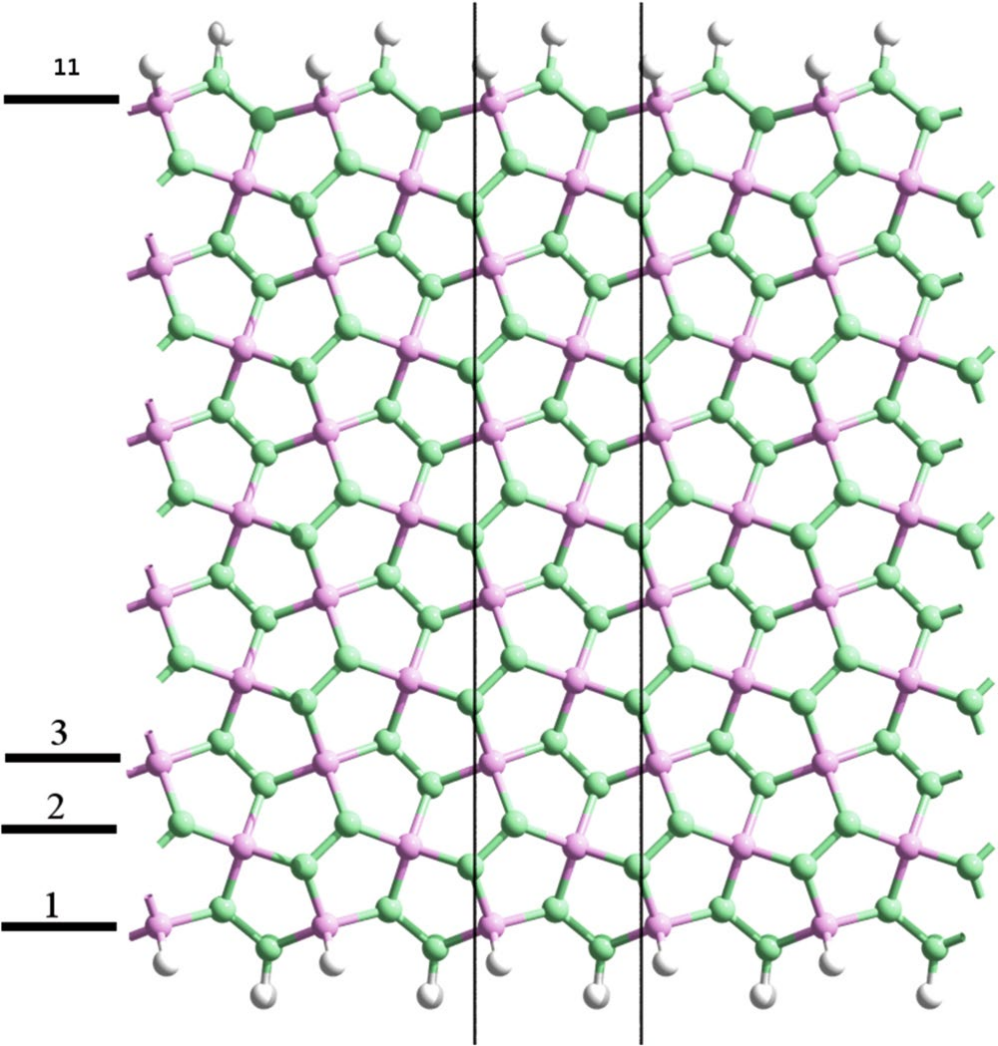
11-PGNR lattice structure. The translational unit cell is marked between two black lines. The labeling based in longitudinal chains is herein illustrated.

In this section we present the band structures and optical absorption spectra of a particular type of penta-graphene nanoribbons (PGNRs) as a means to test our model in nanostructured systems. In particular, we choose PGNRs with sawtooth-like edges^22^, shown in Fig. 4. The reason for this choice is that PGNRs with such edges are not magnetic^23,24^, so we can concentrate in the validity of the tight-binding parameterization focusing on size effects. PGNRs are labeled with the number of longitudinal chains across its width. For example, Fig. 4 depicts a 11-PGNR. Obviously, the symmetry of PGNRs is reduced with respect to PG. Since nanoribbons have translational symmetry in only one direction, we have to resort to the so called rod groups to describe their symmetry. The PGNRs studied in this work belong to the rod group labeled *P*112_1_^28,37^. It has the identity transformation plus a *C*_2_ rotation around the periodic axis of the ribbon combined with a glide plane translation by 1/2*a*, where *a* is the lattice constant vector in the direction with translational symmetry.

#### Band structure of PGNRs

Ultranarrow nanoribbons present strong lattice relaxation effects, so we focus on wider ribbons, for which such effects are not so important and can be described with a small modification of the hopping parameters at the edges. The translational unit cell employed for the calculations is marked with two black lines in Fig. 4. We use the TB parameterization of Table 1 and a hard wall boundary condition for the edges, which means that the dangling bonds of the edge atoms are modeled by hopping matrix elements set to zero. In fact, this is analogous to the inclusion of hydrogen atoms at the edges, but more economical from the computational viewpoint. Since these unsaturated orbitals produce deformations at the edge bonds, we change the corresponding hoppings, increasing their value in 10% in order to mimic such geometrical changes.

**Figure 5:**
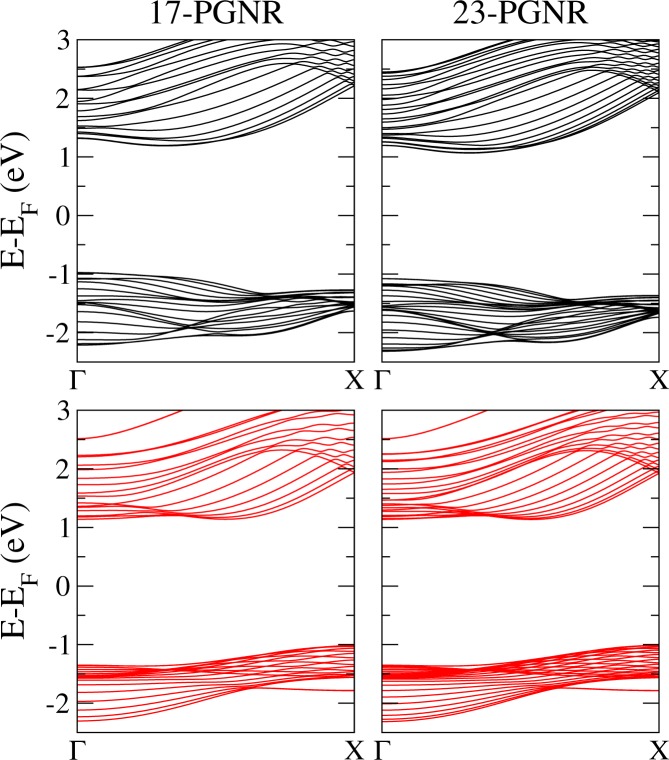
Energy band structures for 17-PGNR and 23-PGNR, as labeled in the Figure. Top panels (black lines) are computed with SIESTA; bottom panels (red lines) are the TB results.

Figure 5 shows the band structures calculated with SIESTA and TB for two particular nanoribbons, namely, 17-PGNR and 23-PGNR, respectively. The band structures obtained by both, the ab-initio and the TB method, show a good agreement. Most remarkably, the low-energy conduction subbands show the indirect minima appearing in the ΓX line. Valence subbands lack some of the fine details concerning some accidental degeneracies that occur along the aforementioned ΓX line. These differences can be easily understood, since the band structure of bulk PG did not reproduce accurately the valence bands of the DFT calculation.

On the other hand, since the ribbons have a lower symmetry with respect to the bulk structure, we expect some decrease in the degeneracy at high symmetry points in the Brillouin zone. This can be seen at Γ and X, where subbands tend to avoid degeneracy in contrast to the case of the bulk. This is observed in both calculations, DFT and TB.

#### Optical absorption of PGNRs

We have additionally computed the optical absorption for nanoribbons with different widths, namely, 17-PGNR to 23-PGNR. In this case we consider that the electric field of the EM radiation oscillates along the nanoribbon axis. In order to make a comparison between both approaches and test the TB parameterization, we have also employed a first-principles method. The optical absorption is also computed employing Eq. (1) using the same external electric field configuration, *k*-space grid and energy broadening in the TB calculation as in the DFT. The included nanoribbon subbands are those stemming from the bulk bands considered for the monolayer optical absorption.

**Figure 6:**
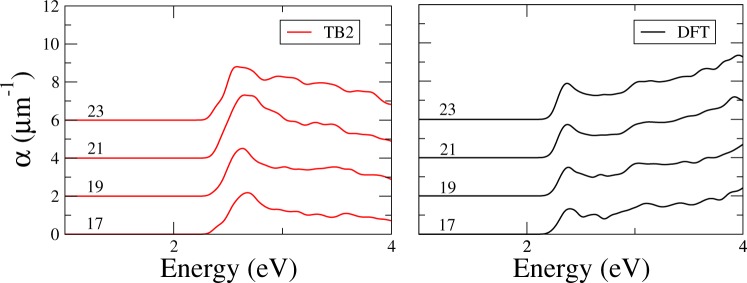
Optical absorption for 17- to 23-PGNRs calculated with (**a**) TB; and (**b**) DFT (SIESTA). For the sake of clarity each curve is shifted a fixed amount.

Results for optical absorption spectra in both approaches are presented in Fig. 6. For the sake of comparison, we restrict the spectra up to 4 eV, given that the DFT peak at this energy is not well reproduced in the TB calculation.

There is a very good overall agreement in the visible and near ultraviolet photon energy range in both, TB and DFT spectra, quantum size effects can be observed as smooth ripples in the spectra. In fact, as the width of the ribbon increases, the features corresponding to the 2D peak at 3 eV emerge more clearly. In view of the validity of the TB parameterization, the description of the optical properties is quite good. In particular, the evolution of the low-energy peak with the width of the nanoribbon is correctly described.

## Conclusions

We have presented a tight-binding parameterization for penta-graphene that provides a very good description of the opto-electronics properties of this material, as it can be seen by comparing the tight-binding calculated magnitudes to the first-principles results. Our choice of parameters was guided by the existence of two types of hybridization in PG: we assigned different parameters to atoms with different hybridizations, and set a geometric cutoff corresponding to third-nearest neighbor interactions for the C2 atoms. The validity of the basis and parameterization was substantiated by the orbital-resolved DFT calculated density of states of PG and by the agreement of the energy bands and optical spectrum calculated within the TB and the DFT approaches, respectively. This parameterization was also employed to model PG nanoribbons with non-magnetic edges, achieving a good description of the quantum-size effects and the recovery of bulk features with increasing widths. We additionally performed a symmetry analysis of the bands, identifying the space group structure of PG and elucidating the contributions of distinct states to the prominent peaks of the optical spectra. Our parameterization can be of interest to model further physical properties of pentagraphene-based nanostructures, for which a first-principles approach is computationally unaffordable.

## Electronic supplementary material

Supplementary information
