# Supplementary material for: Tight-binding model for opto-electronic properties of penta-graphene nanostructures
Source: Sci Rep. 2018 Jul 23;8:11070. doi: 10.1038/s41598-018-29288-8 (PMC6056545; doi:10.1038/s41598-018-29288-8)
Supplement: Supplementary file 1 — Supplementary information [file 41598_2018_29288_MOESM1_ESM.pdf]

Supplementary information to ”Tight-binding model for opto-electronic properties of penta-graphene nanostructures”

Sergio Bravo[1], Julián Correa[2], Leonor Chico[3], and Mónica Pacheco[1,*]

[1]Universidad Técnica Federico Santa María, Departamento de Física, Valparaíso, Casilla 110-V, Chile.
[2]Universidad de Medellín, Facultad de Ciencias Básicas, Medellín, Colombia
[3]Materials Science Factory, Instituto de Ciencia de Materiales de Madrid, Consejo Superior de Investigaciones Científicas, C/ Sor Juana Inés de la Cruz 3, 28049 Madrid, Spain
[*]monica.pacheco@usm.cl

ABSTRACT

In this supplementary information we include the details of the procedure followed to obtain the parameters presented in the main article, as well as two additional examples of tight-binding parameterizations for penta-graphene that give very good descriptions of the bands, without the corrections performed with the aim of reproducing the optical spectrum.

Fitting procedure

We start by performing a fit of the bands along the high symmetry lines. No constraints are imposed on the parameters and 8 bands are fitted by a least squares method. This yields a parameter set that we call TB1, given in Table S1. The corresponding bands are shown in Fig. S1 in blue, along with the DFT bands, plotted as a reference. With this set we start a new fit of the parameters, now in a two-dimensional (2D) grid over the entire Brillouin zone. This is done incrementally: first we take a 2000 k-point grid to adjust the bands, and we increase successively to 4000 and 8000 k-points, which were enough to achieve convergence to the energy dispersion relations; see Fig. S1 in magenta. With this set, that we call TB2 (see Table S2) we start a manual correction procedure with the aim of describing the optical properties. With this purpose, we use the total DOS as a guide to elucidate the main contributions to the optical spectra.

It can be seen that both sets of parameters allow to obtain a good description of the four bands closer to the gap. In fact, the conduction bands are specially well described, presenting the minima along the ΓX and ΓM directions, as in the SIESTA results. However, the valence bands are not so well described, particularly below -3 eV. Even the two highest valence bands are not well reproduced. The undulations of the DFT valence bands produces contributions to the optical spectra that cannot be reproduced with these TB parameterizations, as it can be observed in Fig. S2, where we present the absorption coefficient calculated with the sets TB1 and TB2, as well as with the SIESTA code. As discussed in the main text, we find that the optimal procedure is to correct the parameter set in order to attain the best agreement to the optical spectrum.

Notwithstanding, the description of the lowest conduction bands is so remarkable that we consider them to be of interest for the study of other physical properties, for example, the study of transport in n-doped PG, so we have included them in this Supplementary Information.

E_s^{C1}	E_p^{C1}	E_s^{C2}	E_p^{C2}
-19.99	-6.423	-5.316	7.223

$V_{ss\sigma}^{C1C2}$	$V_{sp\sigma}^{C1C2}$	$V_{pp\sigma}^{C1C2}$	$V_{pp\pi}^{C1C2}$	$V_{ss\sigma}^{C2C2}$	$V_{sp\sigma}^{C2C2}$	$V_{pp\sigma}^{C2C2}$	$V_{pp\pi}^{C2C2}$	$V_{ss\sigma}^{C'2C2}$	$V_{sp\sigma}^{C'2C2}$	$V_{pp\sigma}^{C'2C2}$	$V_{pp\pi}^{C'2C2}$
-8.143	4.593	3.924	-0.162	-11.787	-7.348	11.207	-2.832	-2.478	-0.037	-4.058	-0.722

Table 1. Set TB1 of Slater-Koster tight-binding parameters (in eV) for PG.

E_s^{C1}	E_p^{C1}	E_s^{C2}	E_p^{C2}
-6.433	-4.311	-2.081	7.006

$V_{ss\sigma}^{C1C2}$	$V_{sp\sigma}^{C1C2}$	$V_{pp\sigma}^{C1C2}$	$V_{pp\pi}^{C1C2}$	$V_{ss\sigma}^{C2C2}$	$V_{sp\sigma}^{C2C2}$	$V_{pp\sigma}^{C2C2}$	$V_{pp\pi}^{C2C2}$	$V_{ss\sigma}^{C'2C2}$	$V_{sp\sigma}^{C'2C2}$	$V_{pp\sigma}^{C'2C2}$	$V_{pp\pi}^{C'2C2}$
-3.555	2.246	3.903	-0.262	-11.731	-10.017	16.589	-1.862	-2.504	-1.080	-3.447	0.921

Table 2. Set TB2 of Slater-Koster tight-binding parameters (in eV) for PG.

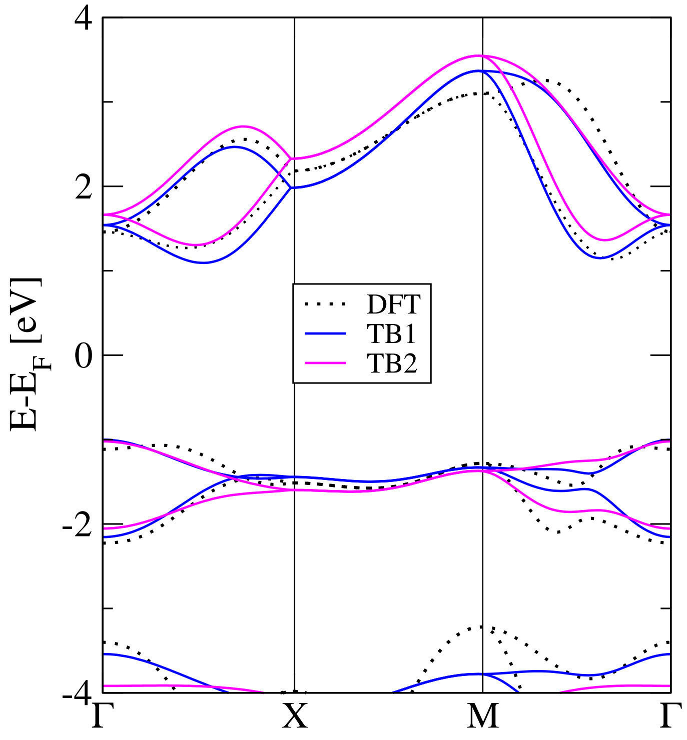

Figure 1. PG energy band structure near the Fermi level calculated with DFT (black dotted lines), and the two TB parameters sets, TB1 (blue lines) and TB2 (magenta lines).

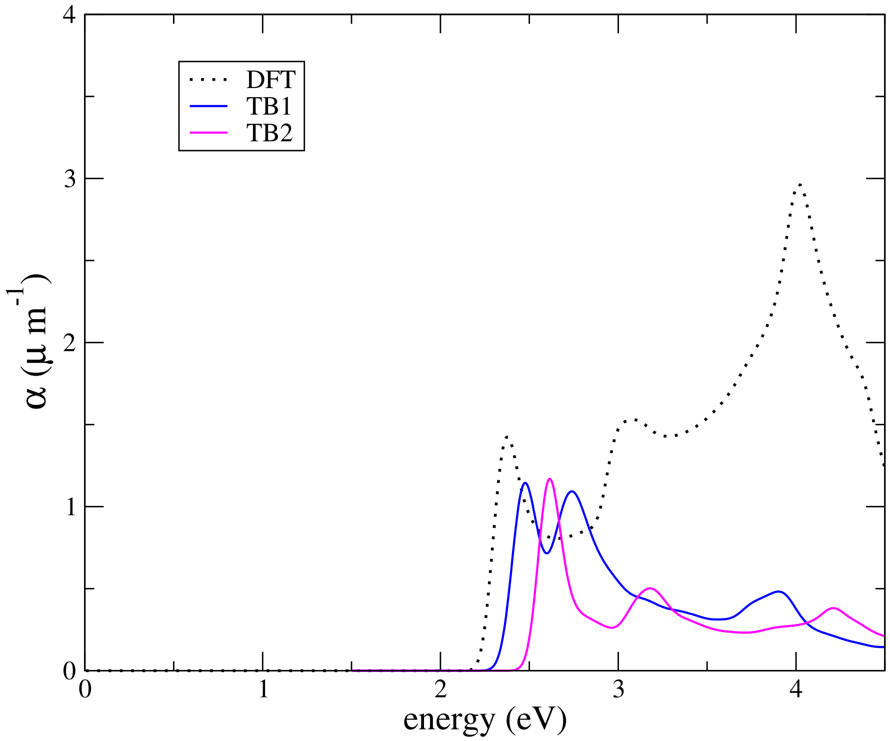

Figure 2. Optical absorption for PG calculated with DFT (black dotted lines) and with the two tight-binding parameterizations TB1 (blue lines) and TB2 (magenta lines).
